# Supplementary material for: The RNA Methyltransferase NSUN2 and Its Potential Roles in Cancer
Source: Cells. 2020 Jul 22;9(8):1758. doi: 10.3390/cells9081758 (PMC7463552; doi:10.3390/cells9081758)
Supplement: Supplementary file 1 [file cells-09-01758-s001.zip › Supplementary Figure 1 R1.pdf]

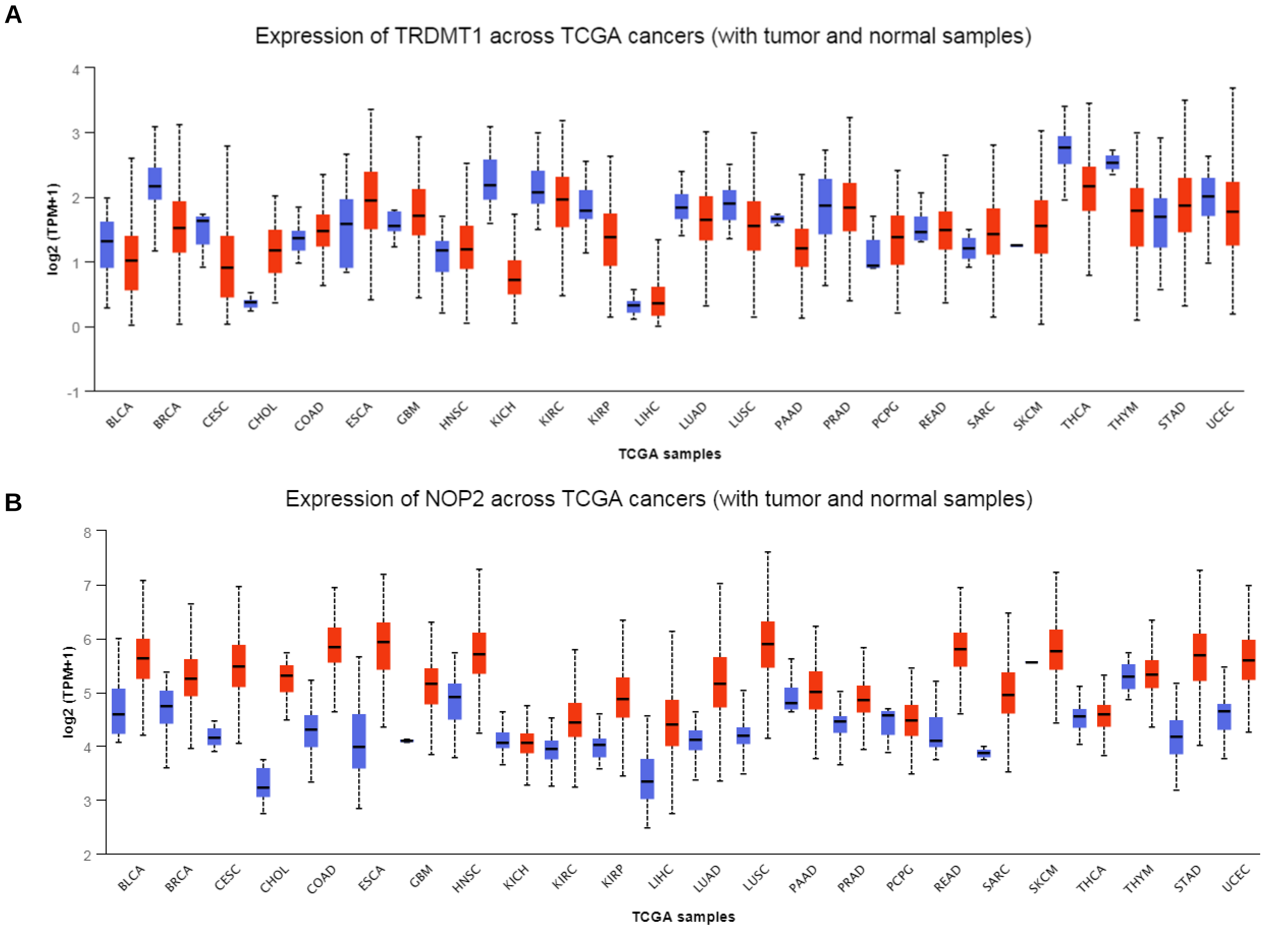

Supplementary Figure 1. Pan Cancer Expression of various RNMTs

C

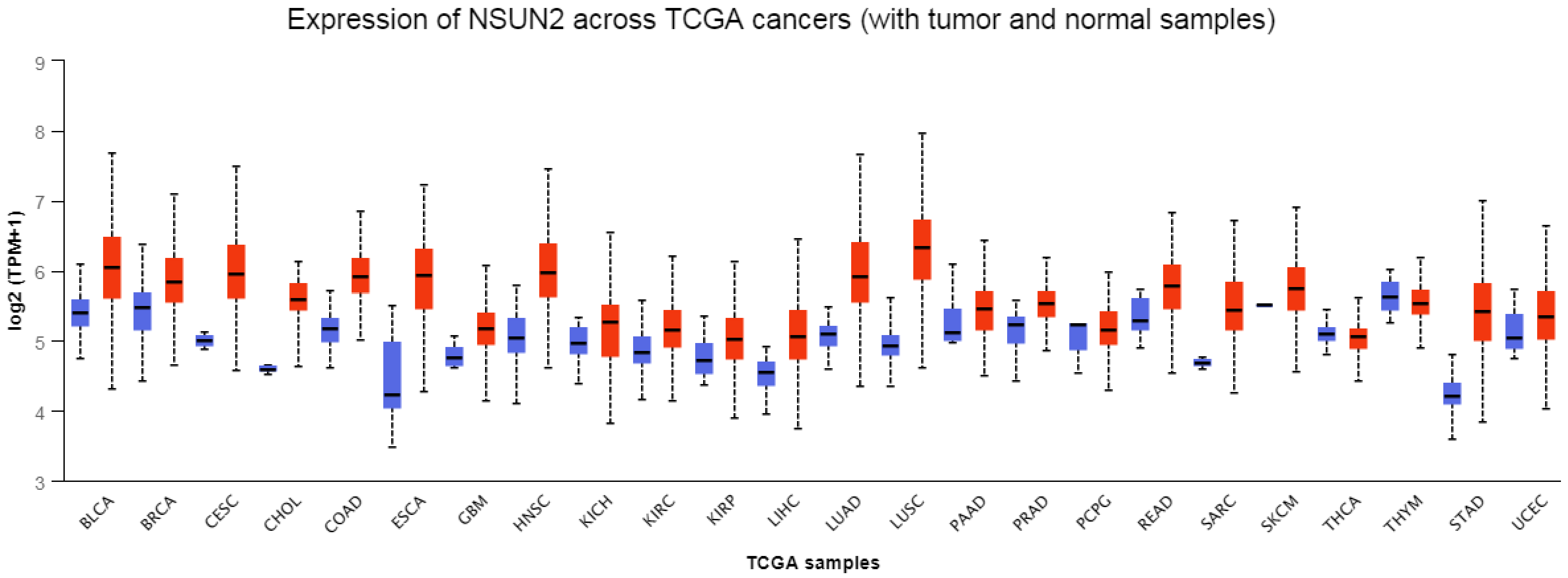

D

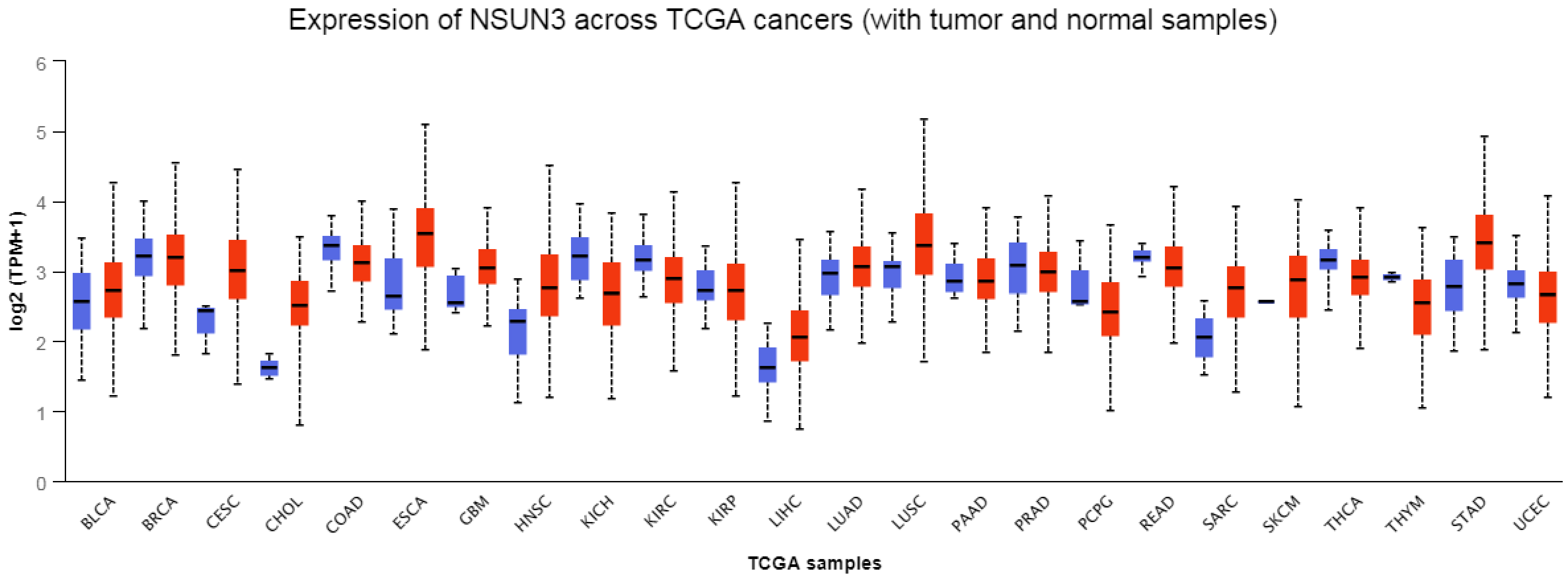

Supplementary Figure 1. Pan Cancer Expression of various RNMTs

E

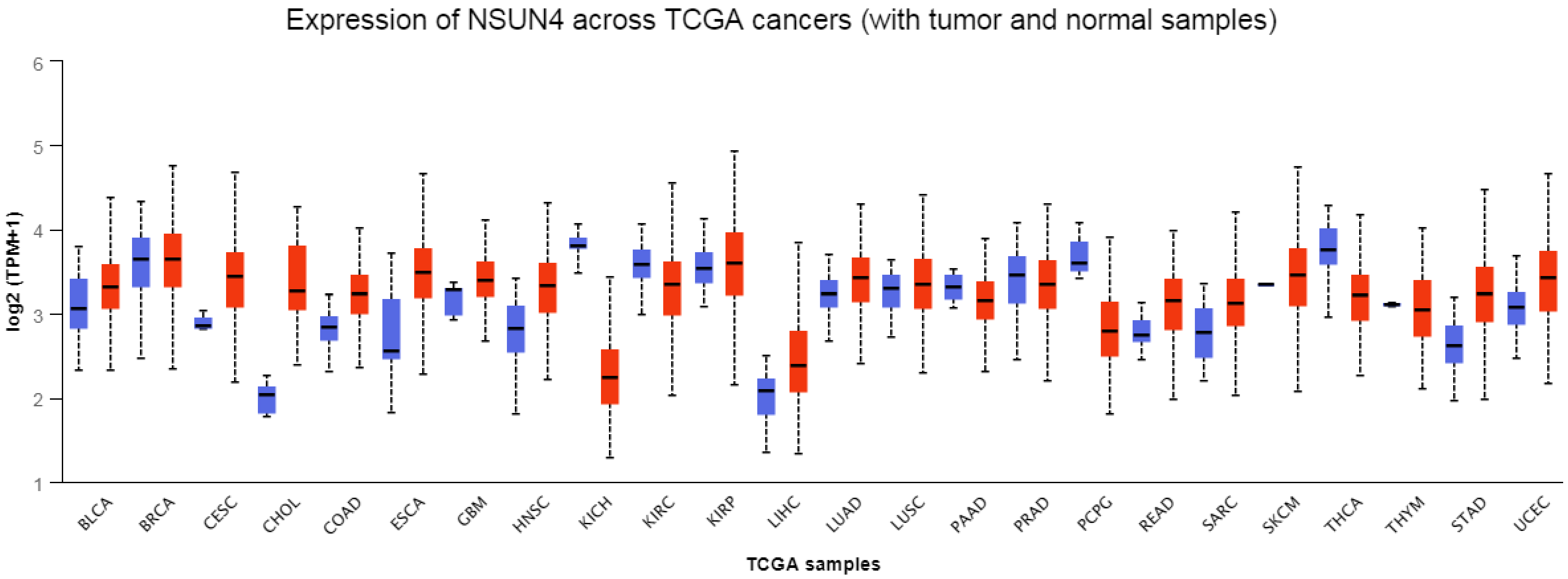

F

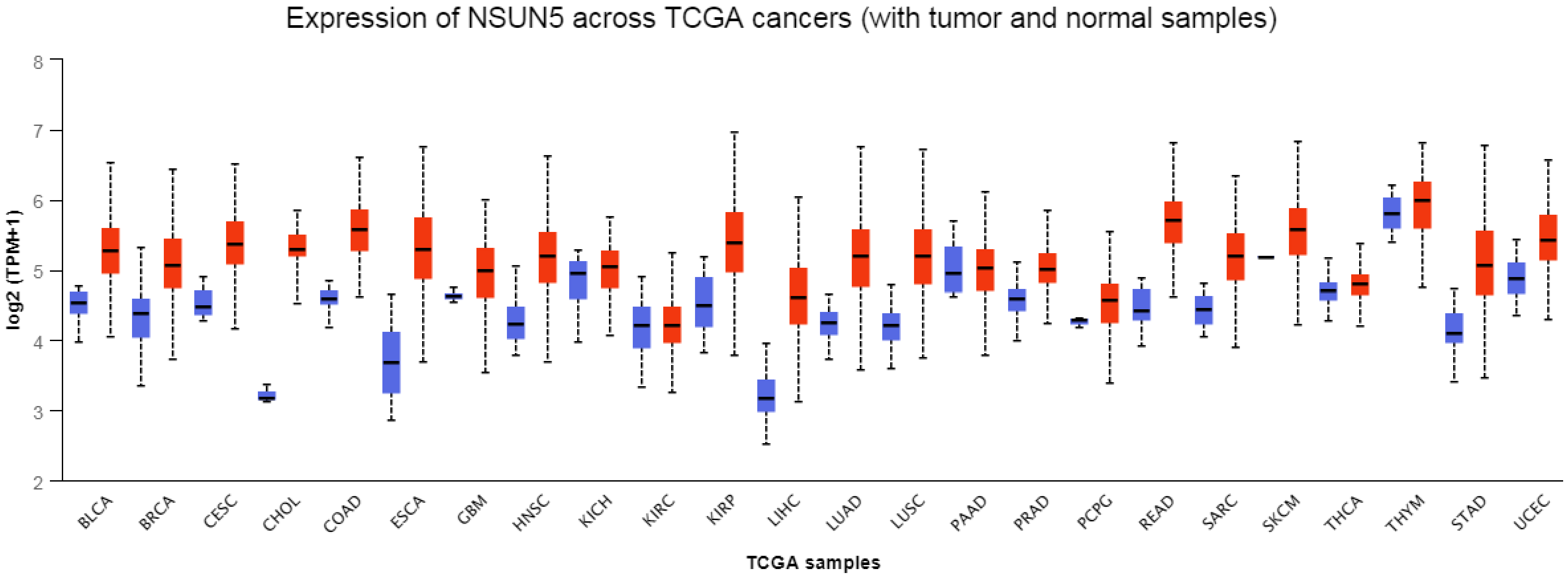

Supplementary Figure 1. Pan Cancer Expression of various RNMTs

G

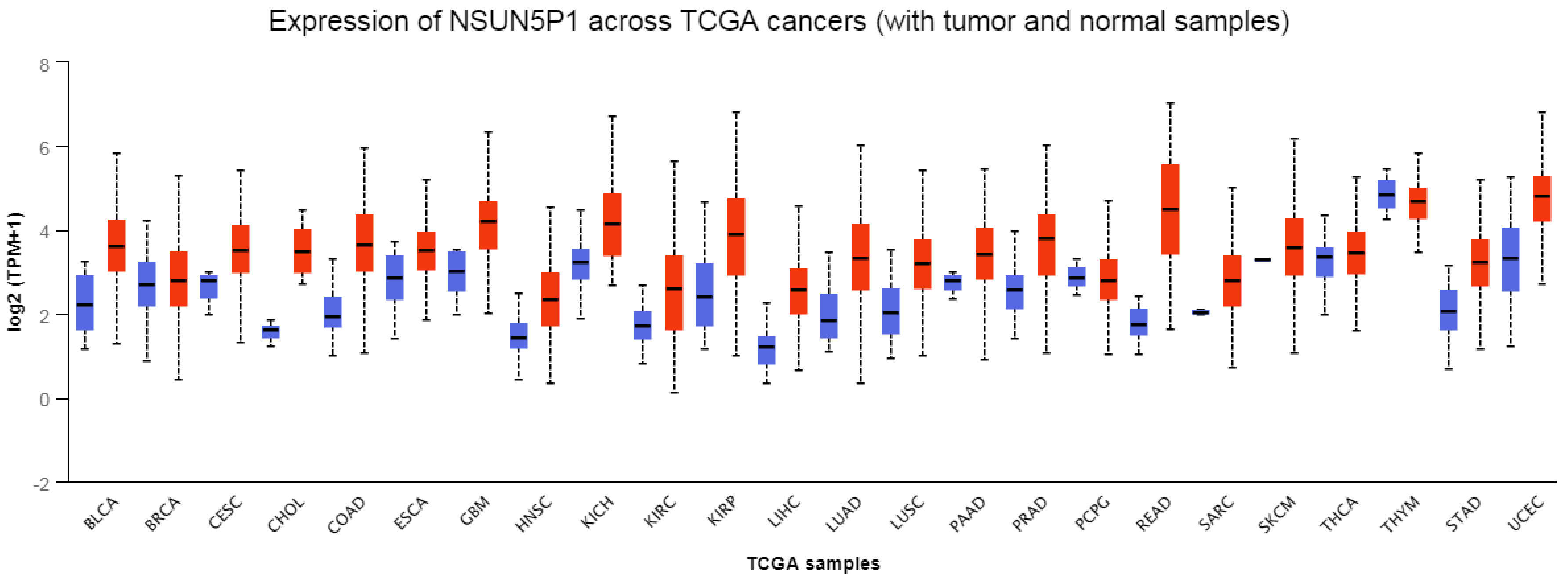

H

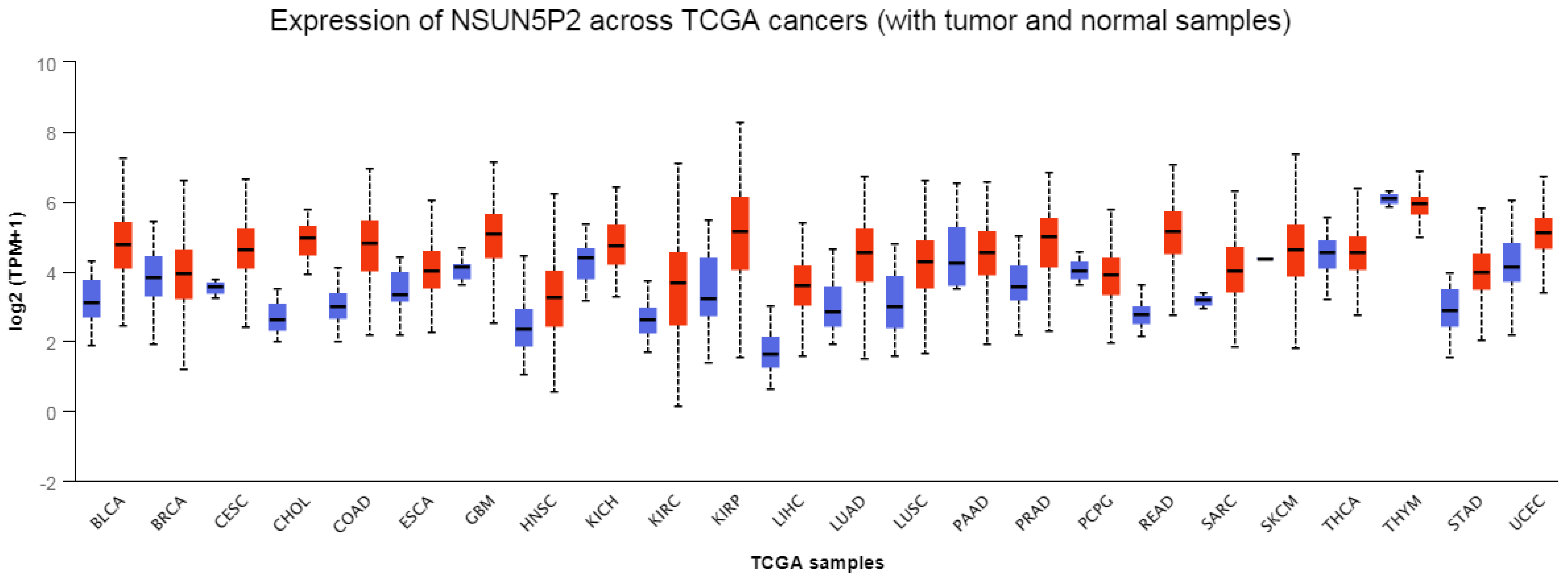

Supplementary Figure 1. Pan Cancer Expression of various RNMTs

I

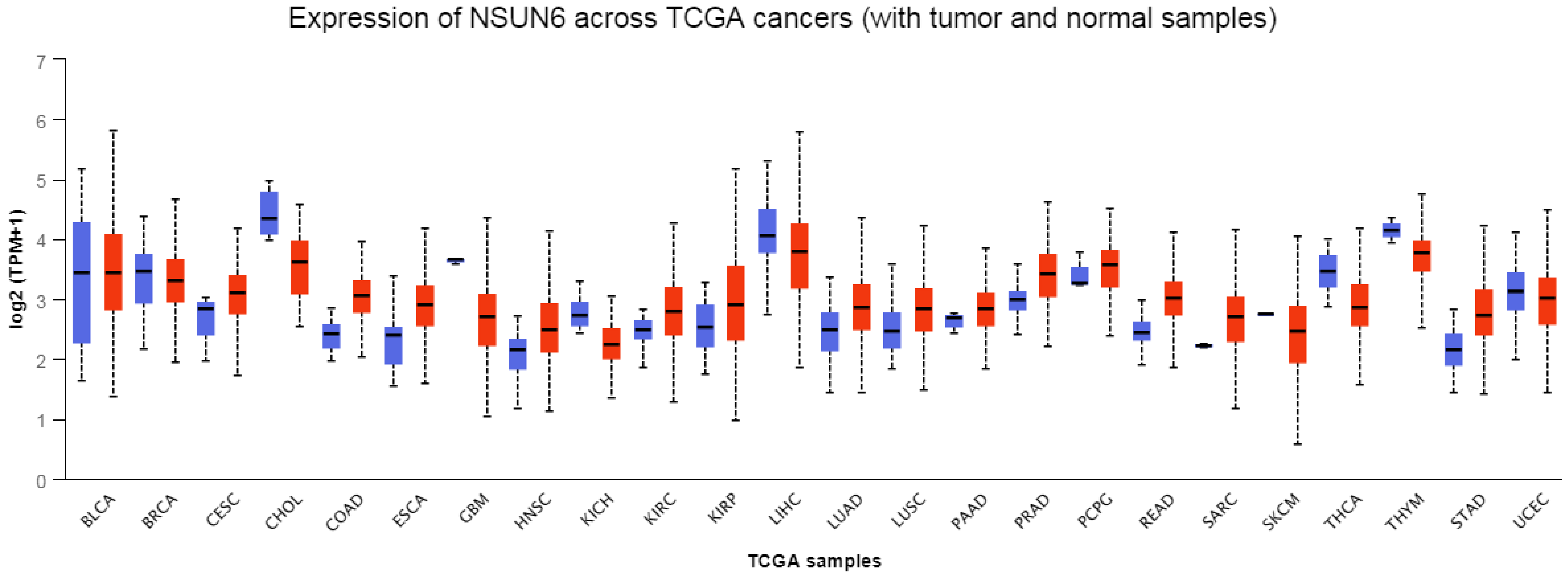

J

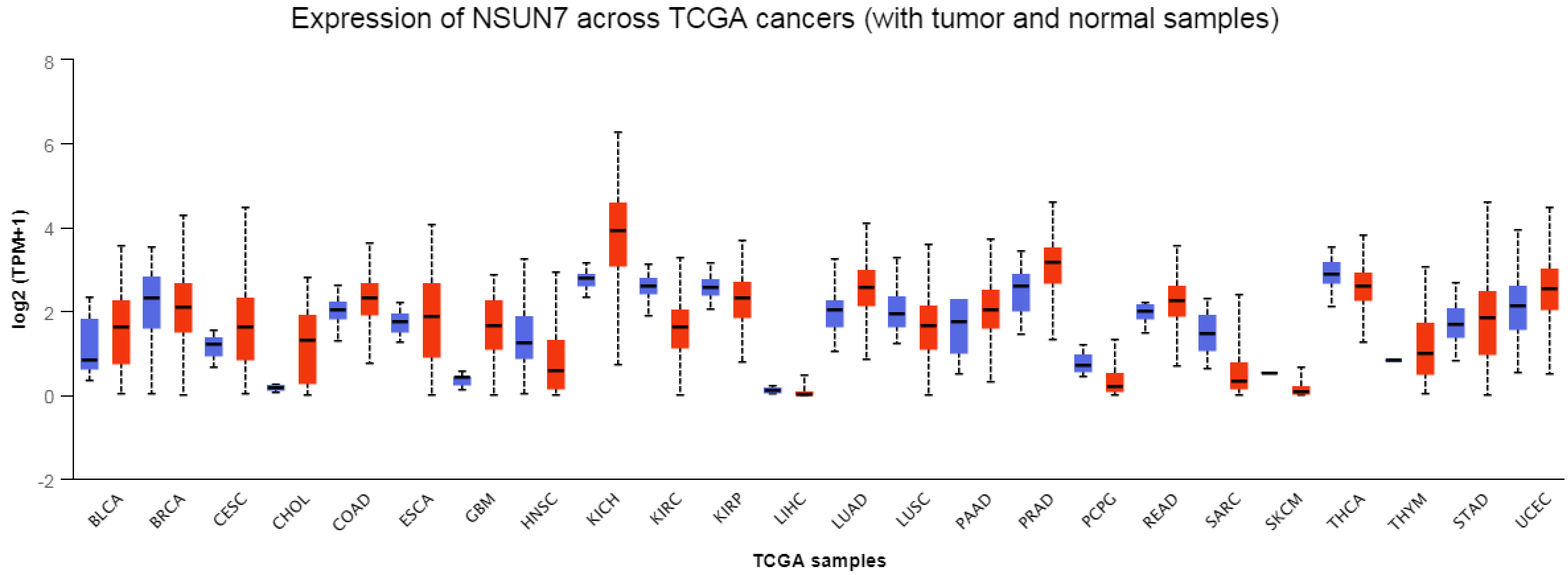

Supplementary Figure 1. Pan Cancer Expression of various RNMTs
