# Supplementary material for: The RNA Methyltransferase NSUN2 and Its Potential Roles in Cancer
Source: Cells. 2020 Jul 22;9(8):1758. doi: 10.3390/cells9081758 (PMC7463552; doi:10.3390/cells9081758)
Supplement: Supplementary file 1 [file cells-09-01758-s001.zip › Supplementary Figure 2 R1.pdf]

Supplementary Figure 2 – Stratified analysis of LUAD and LUSC datasets using UALCAN – Normal vs Tumor

A

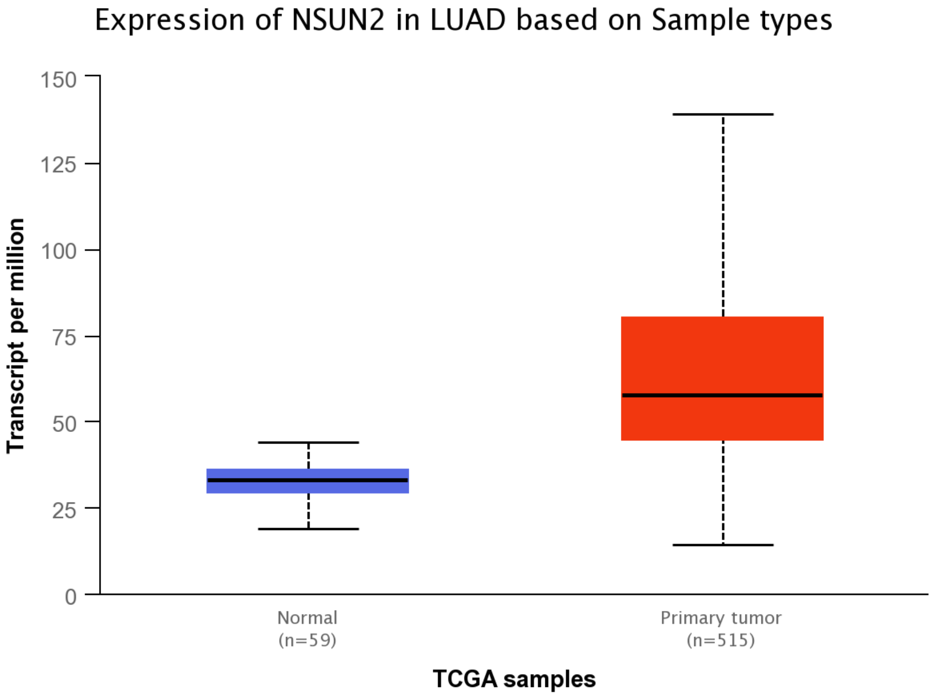

Comparison	Statistical significance
Normal-vs-Primary	1.62436730732907E-12

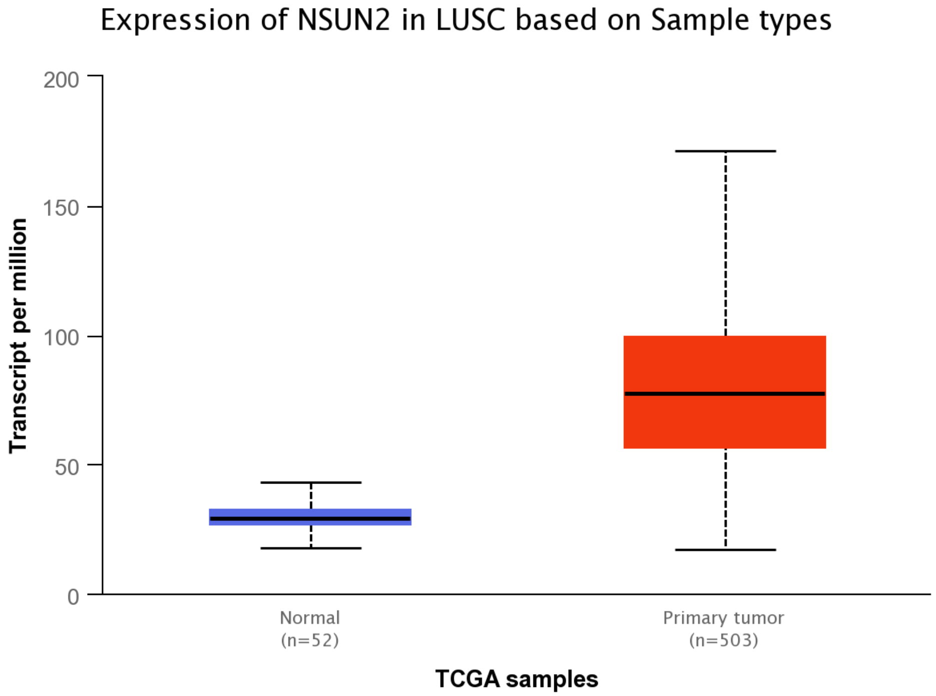

Comparison	Statistical significance
Normal-vs-Primary	1.62447832963153E-12

Supplementary Figure 2 – Stratified analysis of LUAD and LUSC datasets using UALCAN – Cancer Stage

B

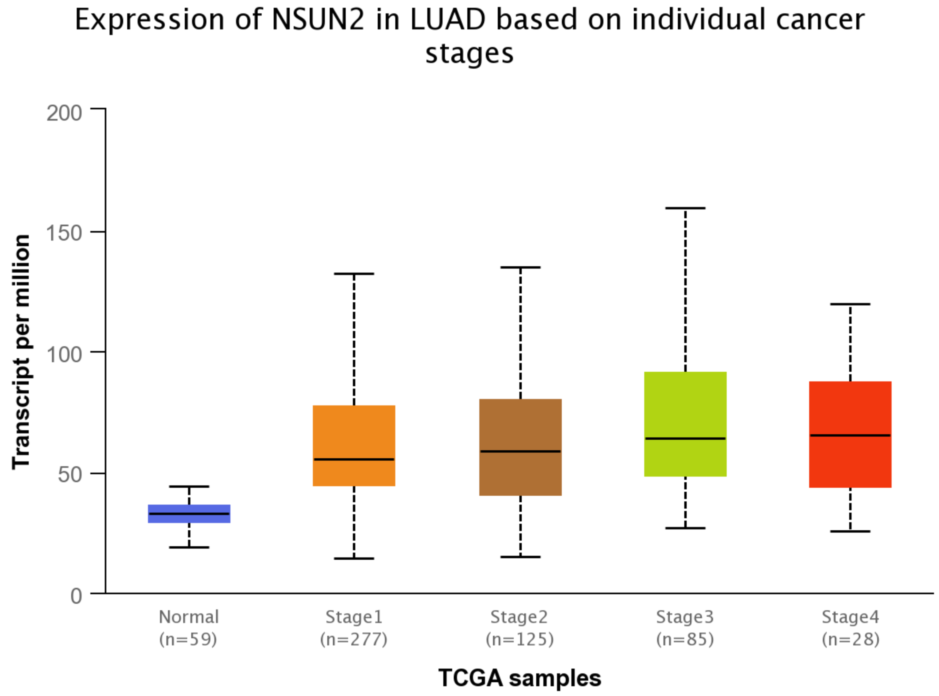

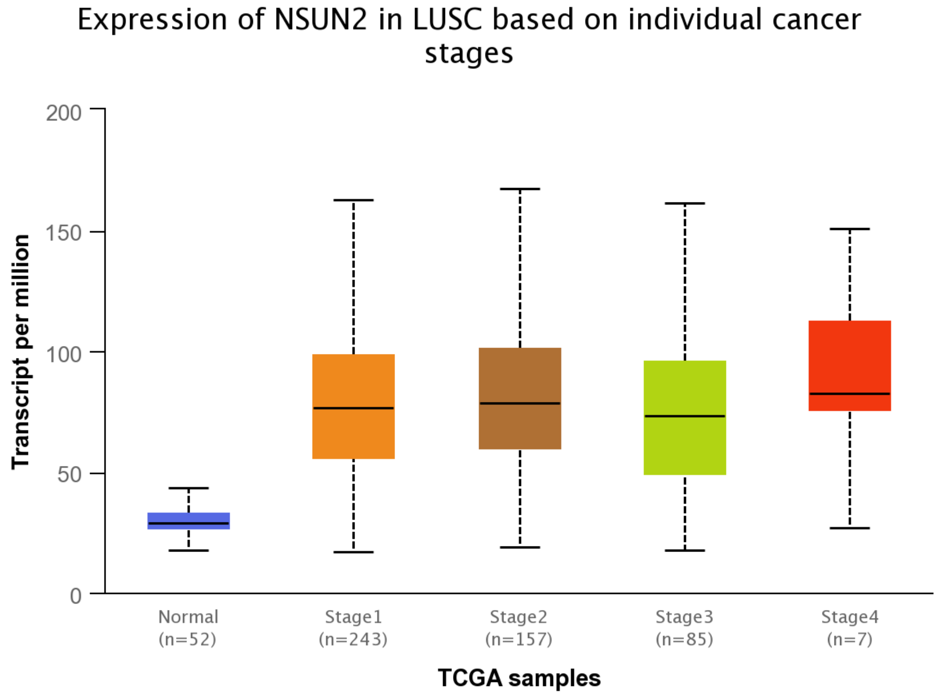

Comparison	Statistical significance
Normal-vs-Stage1	1.62447832963153E-12
Normal-vs-Stage2	<1E-12
Normal-vs-Stage3	5.55111512312578E-16
Normal-vs-Stage4	3.9552999997472E-07
Stage1-vs-Stage2	3.834400E-01
Stage1-vs-Stage3	7.104500E-02
Stage1-vs-Stage4	5.890200E-01
Stage2-vs-Stage3	4.370800E-01
Stage2-vs-Stage4	9.927400E-01
Stage3-vs-Stage4	6.028800E-01

Comparison	Statistical significance
Normal-vs-Stage1	<1E-12
Normal-vs-Stage2	<1E-12
Normal-vs-Stage3	<1E-12
Normal-vs-Stage4	6.836100E-03
Stage1-vs-Stage2	6.048200E-01
Stage1-vs-Stage3	2.794600E-01
Stage1-vs-Stage4	8.855400E-01
Stage2-vs-Stage3	4.205600E-01
Stage2-vs-Stage4	7.316200E-01
Stage3-vs-Stage4	5.822000E-01

Supplementary Figure 2 – Stratified analysis of LUAD and LUSC datasets using UALCAN – Patients race

C

Expression of NSUN2 in LUAD based on patient's race

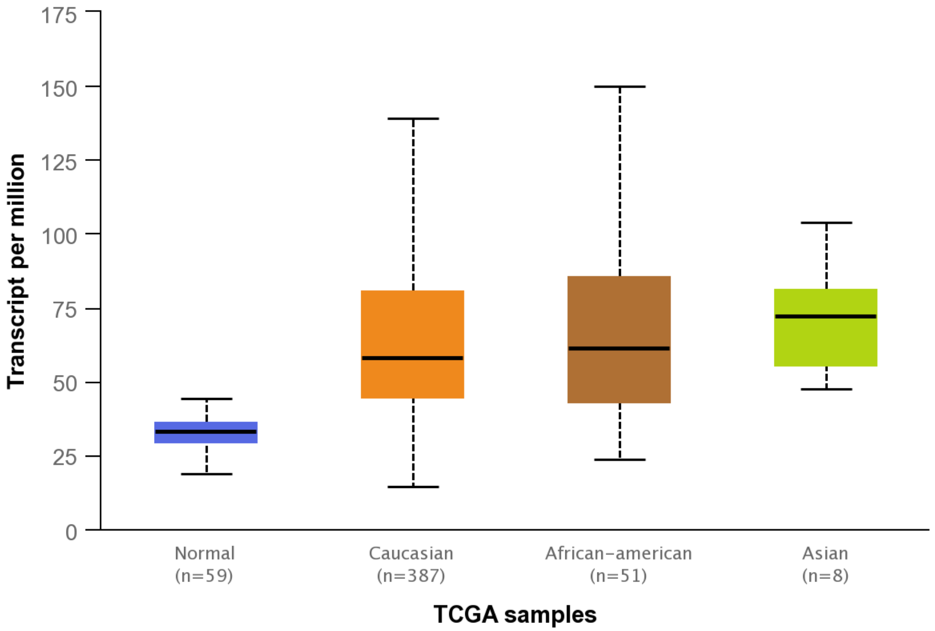

Comparison	Statistical significance
Normal-vs-Caucasian	<1E-12
Normal-vs-AfricanAmerican	1.0816003648273E-10
Normal-vs-Asian	5.024600E-04
Caucasian-vs-AfricanAmerican	6.849600E-01
Caucasian-vs-Asian	9.568000E-01
AfricanAmerican-vs-Asian	8.825000E-01

Expression of NSUN2 in LUSC based on patient's race

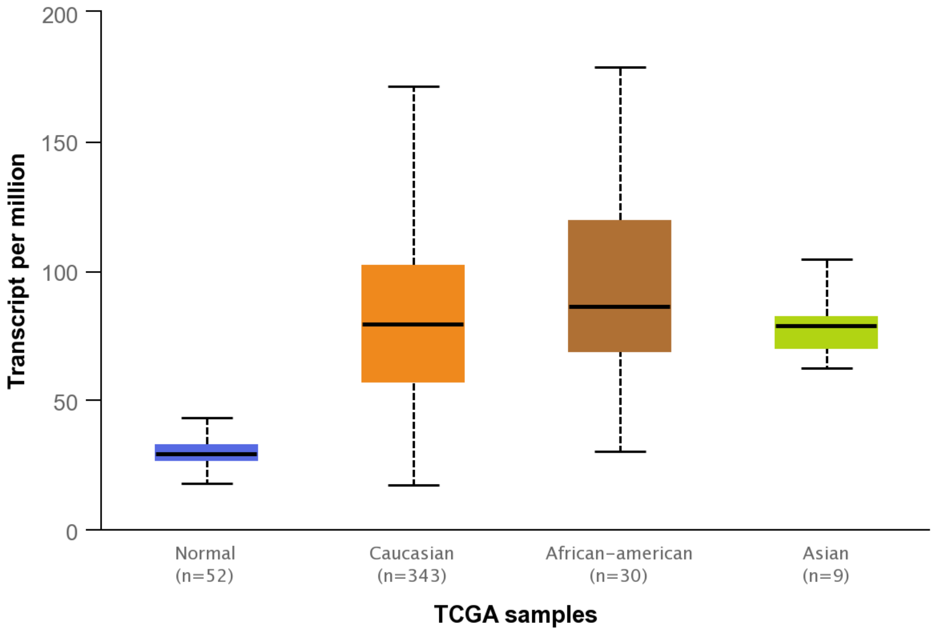

Comparison	Statistical significance
Normal-vs-Caucasian	1.62436730732907E-12
Normal-vs-AfricanAmerican	8.46229752937688E-11
Normal-vs-Asian	2.172600E-04
Caucasian-vs-AfricanAmerican	4.473200E-01
Caucasian-vs-Asian	2.631800E-01
AfricanAmerican-vs-Asian	2.353200E-01

Supplementary Figure 2 – Stratified analysis of LUAD and LUSC datasets using UALCAN – Patient Gender

D

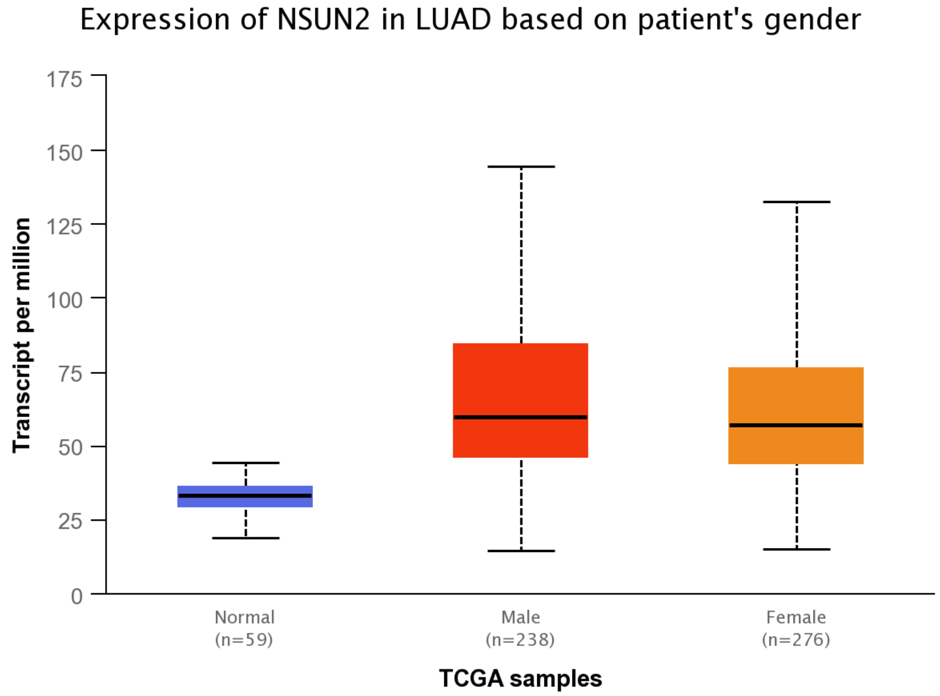

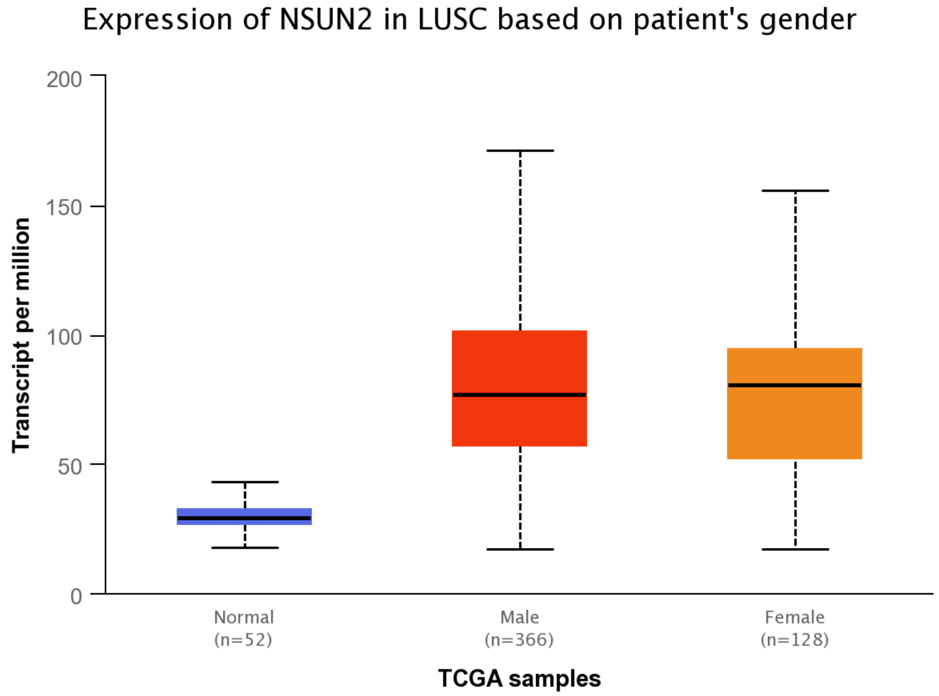

Comparison	Statistical significance
Normal-vs-Male	1.62436730732907E-12
Normal-vs-Female	<1E-12
Male-vs-Female	8.571400E-01

Comparison	Statistical significance
Normal-vs-Male	1.62436730732907E-12
Normal-vs-Female	<1E-12
Male-vs-Female	1.889370E-01

Supplementary Figure 2 – Stratified analysis of LUAD and LUSC datasets using UALCAN – Patient Age

E

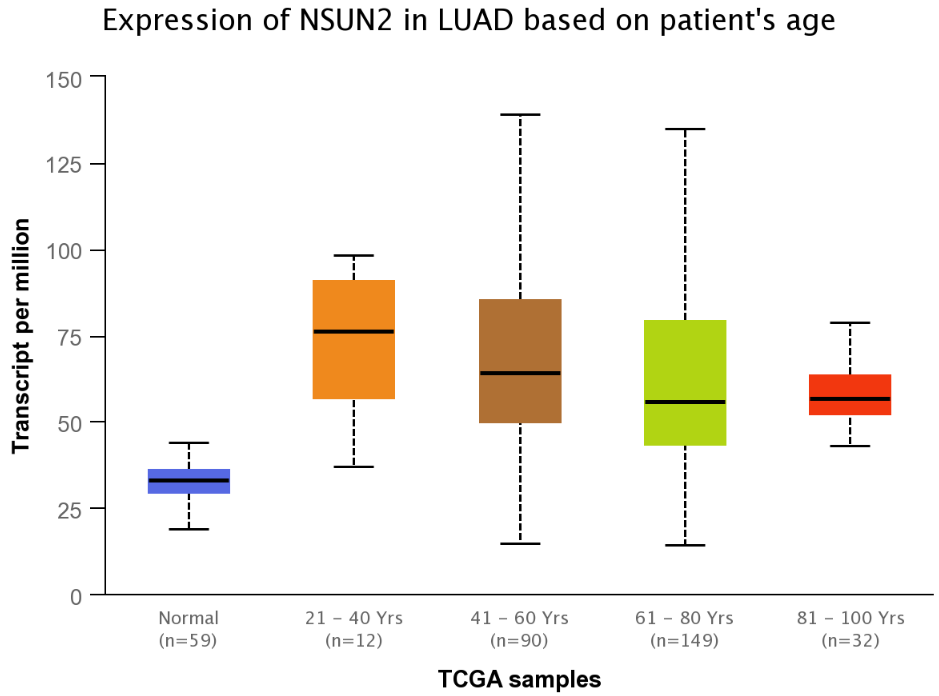

Comparison	Statistical significance
Normal-vs-Age(21-40Yrs)	5.959500E-02
**Normal-vs-Age(41-60Yrs)**	**1.62447832963153E-12**
**Normal-vs-Age(61-80Yrs)**	**1.62436730732907E-12**
**Normal-vs-Age(81-100Yrs)**	**1.83231999999478E-05**
Age(21-40Yrs)-vs-Age(41-60Yrs)	8.044400E-01
Age(21-40Yrs)-vs-Age(61-80Yrs)	8.040600E-01
Age(21-40Yrs)-vs-Age(81-100Yrs)	8.245000E-01
**Age(41-60Yrs)-vs-Age(61-80Yrs)**	**1.545560E-02**
Age(41-60Yrs)-vs-Age(81-100Yrs)	2.926400E-01
Age(61-80Yrs)-vs-Age(81-100Yrs)	9.542000E-01

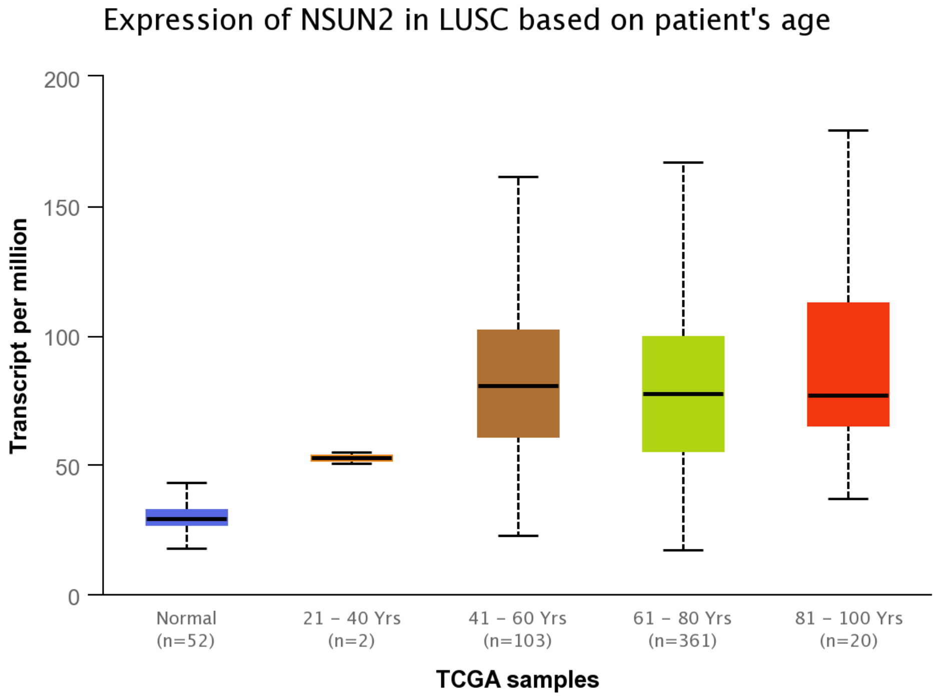

Comparison	Statistical significance
**Normal-vs-Age(21-40Yrs)**	**1.221460E-04**
**Normal-vs-Age(41-60Yrs)**	**<1E-12**
**Normal-vs-Age(61-80Yrs)**	**<1E-12**
**Normal-vs-Age(81-100Yrs)**	**4.31220000000065E-05**
Age(21-40Yrs)-vs-Age(41-60Yrs)	1.945820E-01
Age(21-40Yrs)-vs-Age(61-80Yrs)	2.693200E-01
Age(21-40Yrs)-vs-Age(81-100Yrs)	2.538600E-01
Age(41-60Yrs)-vs-Age(61-80Yrs)	7.510000E-01
Age(41-60Yrs)-vs-Age(81-100Yrs)	1.768010E-01
Age(61-80Yrs)-vs-Age(81-100Yrs)	1.442160E-01

Supplementary Figure 2 – Stratified analysis of LUAD and LUSC datasets using UALCAN – Patient Smoking History

F

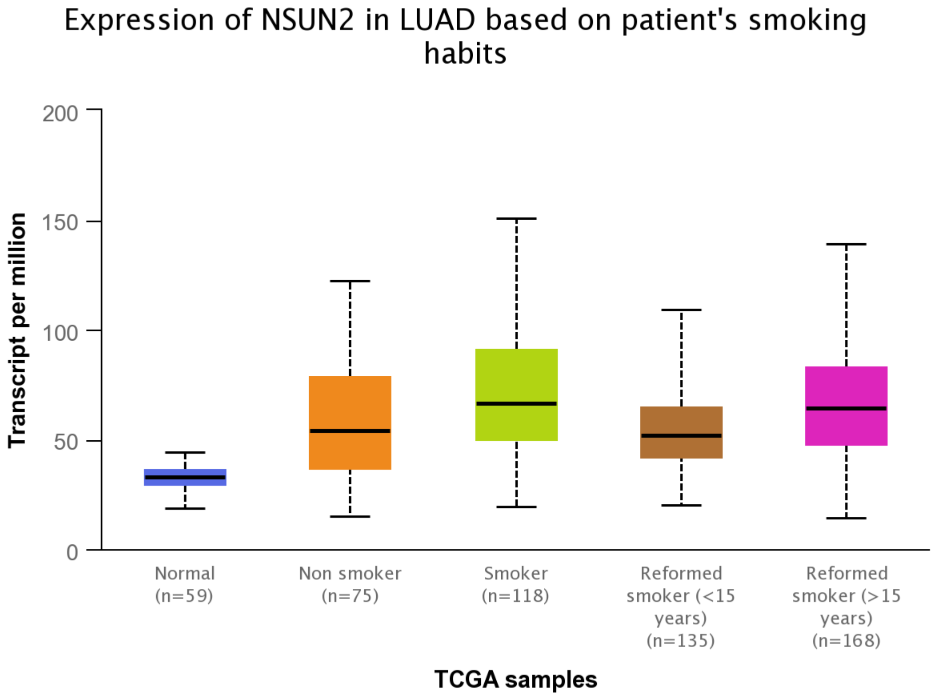

Comparison	Statistical significance
Normal-vs-Non smoker	1.01099995042375E-09
Normal-vs-Smoker	<1E-12
Normal-vs-Reformed smoker1	<1E-12
Normal-vs-Reformed smoker2	<1E-12
Non smoker-vs-Smoker	7.256700E-03
Non smoker-vs-Reformed smoker1	9.729400E-01
Non smoker-vs-Reformed smoker2	3.013900E-02
Smoker-vs-Reformed smoker1	5.585500E-04
Smoker-vs-Reformed smoker2	2.944200E-01
Reformed smoker1-vs-Reformed smoker2	3.335100E-03

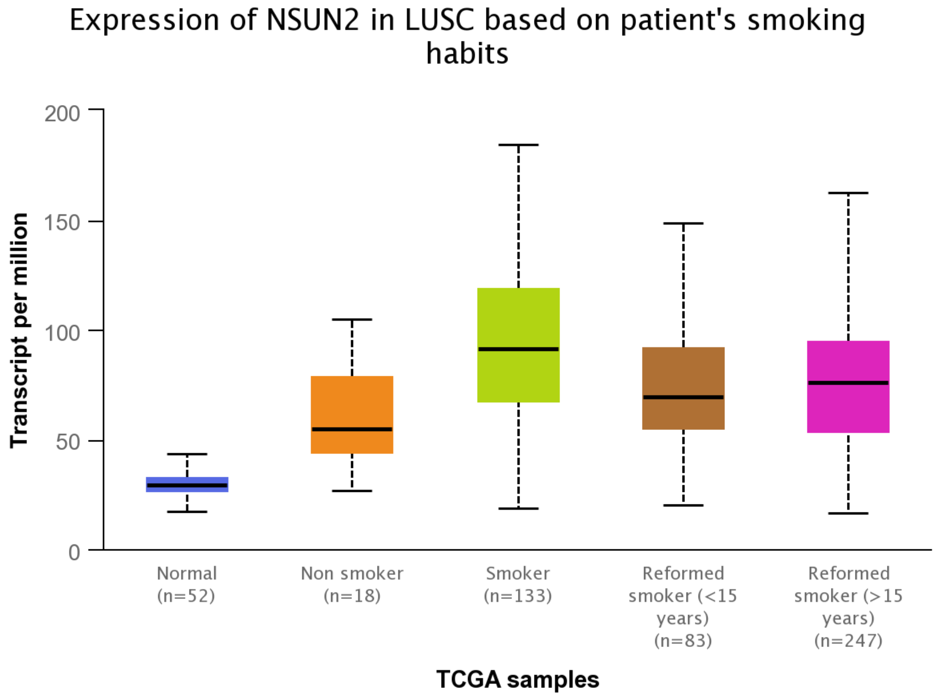

Comparison	Statistical significance
Normal-vs-Non smoker	7.956200E-03
Normal-vs-Smoker	1.62458935193399E-12
Normal-vs-Reformed smoker1	1.62481139653892E-12
Normal-vs-Reformed smoker2	<1E-12
Non smoker-vs-Smoker	1.232400E-01
Non smoker-vs-Reformed smoker1	2.734600E-01
Non smoker-vs-Reformed smoker2	4.526000E-01
Smoker-vs-Reformed smoker1	2.311800E-01
Smoker-vs-Reformed smoker2	4.803300E-03
Reformed smoker1-vs-Reformed smoker2	4.894600E-01

Supplementary Figure 2 – Stratified analysis of LUAD and LUSC datasets using UALCAN – Nodal Metastasis

G

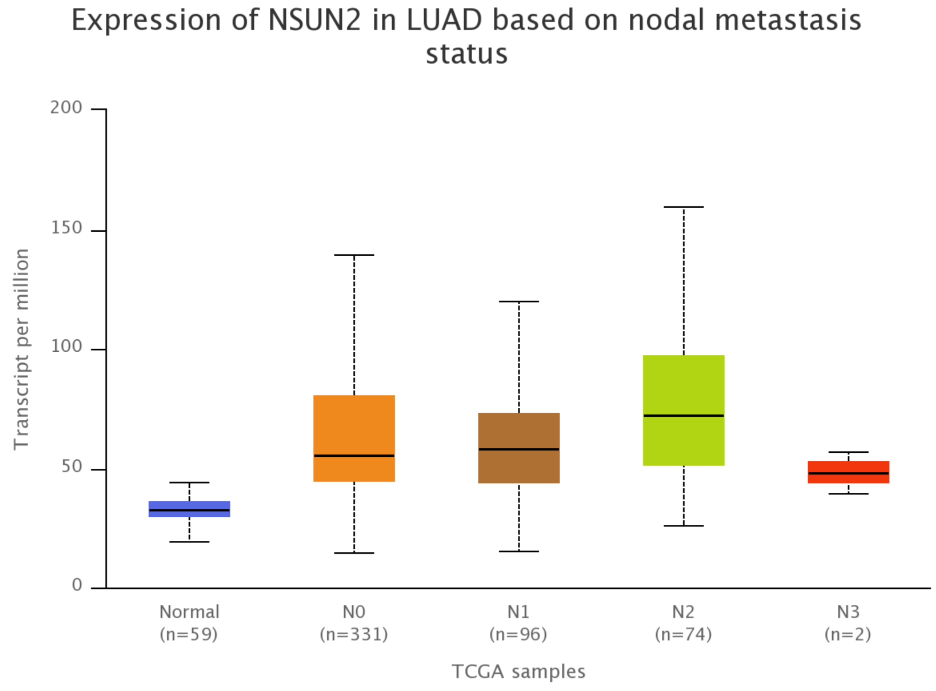

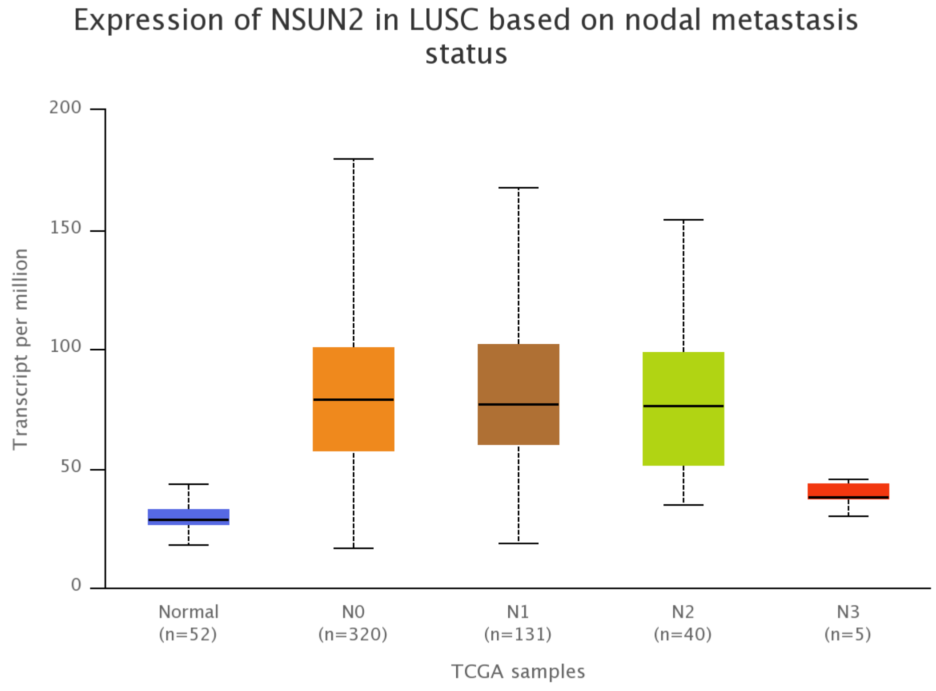

Comparison	Statistical significance
Normal-vs-N0	<1E-12
Normal-vs-N1	1.63025148935958E-12
Normal-vs-N2	1.3988810110277E-14
Normal-vs-N3	7.162500E-04
N0-vs-N1	4.704800E-01
N0-vs-N2	4.265600E-02
N0-vs-N3	4.323600E-01
N1-vs-N2	2.510400E-01
N1-vs-N3	4.409200E-01
N2-vs-N3	3.162400E-01

pathologic_N descriptions

N0	No regional lymph node metastasis	N1	Metastases in 1 to 3 axillary lymph nodes
N2	Metastases in 4 to 9 axillary lymph nodes	N3	Metastases in 10 or more axillary lymph nodes

Comparison	Statistical significance
Normal-vs-N0	1.62447832963153E-12
Normal-vs-N1	<1E-12
Normal-vs-N2	1.63269953112888E-10
Normal-vs-N3	2.656000E-02
N0-vs-N1	1.583410E-01
N0-vs-N2	5.164600E-01
N0-vs-N3	2.39349995379001E-09
N1-vs-N2	9.057800E-01
N1-vs-N3	6.88210044508253E-10
N2-vs-N3	3.33890000092651E-08

Supplementary Figure 2 – Stratified analysis of LUAD and LUSC datasets using UALCAN – p53 Mutation Status

H

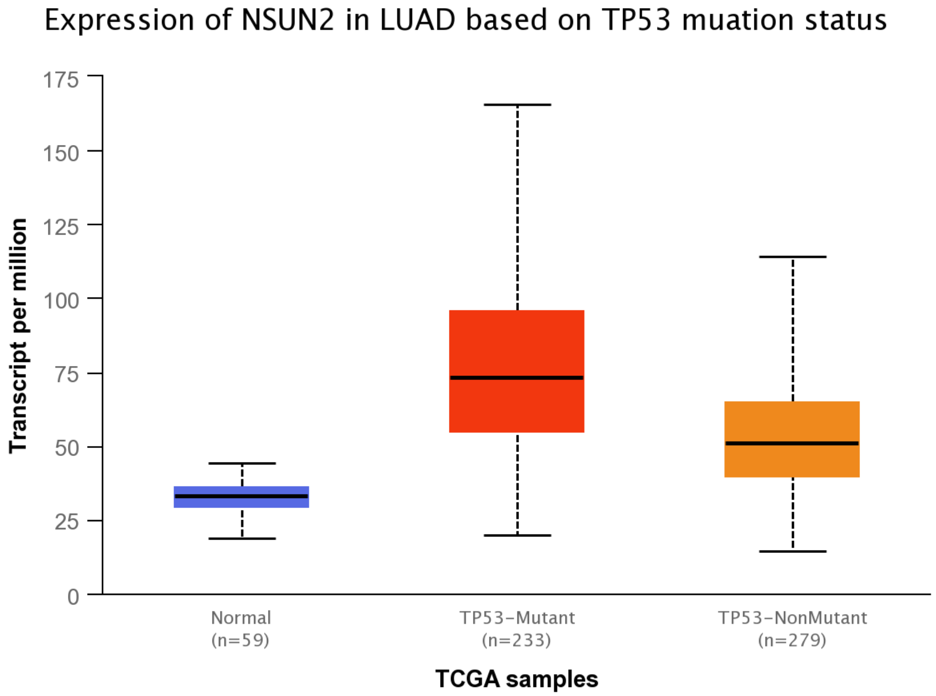

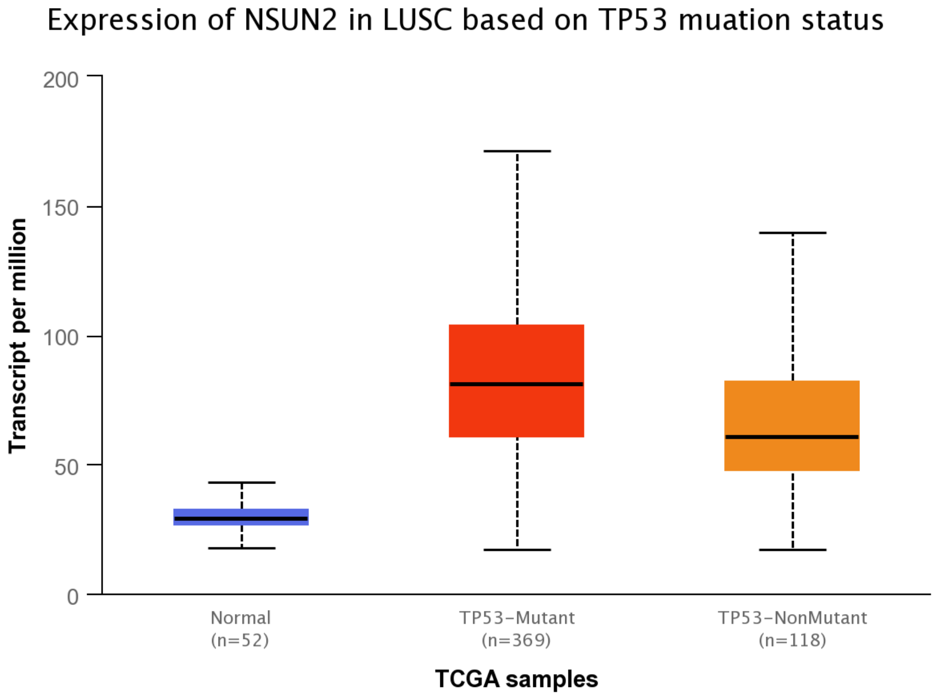

Comparison	Statistical significance
Normal-vs-TP53-Mutant	1.62447832963153E-12
Normal-vs-TP53-NonMutant	1.62436730732907E-12
TP53-Mutant-vs-TP53-NonMutant	1.18460796727504E-13

Comparison	Statistical significance
Normal-vs-TP53-Mutant	<1E-12
Normal-vs-TP53-NonMutant	<1E-12
TP53-Mutant-vs-TP53-NonMutant	2.107700E-02
