# Supplementary material for: The RNA Methyltransferase NSUN2 and Its Potential Roles in Cancer
Source: Cells. 2020 Jul 22;9(8):1758. doi: 10.3390/cells9081758 (PMC7463552; doi:10.3390/cells9081758)
Supplement: Supplementary file 1 [file cells-09-01758-s001.zip › Supplementary Figure Legends R1.docx]

Supplementary Figure Legends

Supplementary Figure S1. Pan-Cancer Expression of TRDMT1 and NOP2 (Normal vs. Tumor)

Pan-Cancer Expression of RNMTs (Normal vs. Tumor) was assessed across all existing TCGA datasets using UALCAN [[94](#_ENREF_94)]. Altered expression (A) TRDMT1; (B) NOP2; (C) NSUN2; (D) NSUN3; (E) NSUN4; (F) NSUN5; (G) NSUN5P1; (H) NSUN5P2; (I) NSUN6 and (J) NSUN7 can be seen across many cancers.

Supplementary Figure S2. Stratified analysis of LUAD and LUSC datasets.

The available LUAD and LUSC TCGA datasets were re-analysed using UALCAN [[94](#_ENREF_94)]. Significant alterations were observed in both datasets for (A) normal vs tumor; (B) tumor stage; (C) race; (D) gender; (E) age; (F) smoking habit; (G) nodal metastasis and (H) p53 mutation status.
